# Association of distance to diagnosis and area-based social measures with stage at diagnosis among Iowans with HPV-related cancers

**DOI:** 10.1007/s10552-025-02066-4

**Published:** 2025-09-10

**Authors:** Emily Janio, Amanda R. Kahl, Dosten Kpozehouen, Natoshia Askelson, Sarah H. Nash

**Affiliations:** 1 College of Public Health, Community and Behavioral Health Department, University of Iowa, Iowa City, IA USA; 2 College of Public Health, Iowa Cancer Registry, Epidemiology Department, University of Iowa, Iowa City, IA USA; 3 College of Public Health, Biostatistics Department, University of Iowa, Iowa City, IA USA

**Keywords:** Cancer registry, Oropharyngeal cancer, Cervical cancer, Community ecological status, Travel time, HPV-associated cancer

## Abstract

**Purpose:**

Human papillomavirus (HPV) causes oral and anogenital cancers, the incidence of which is increasing. Late-stage diagnosis is associated with increased mortality. Neighborhood-level characteristics and distance to place of diagnosis may impact timely diagnosis. Being a largely rural state, Iowa presents a unique location to understand the association between distance, neighborhood characteristics, and stage at diagnosis.

**Methods:**

Data from the Iowa Cancer Registry from 2010 to 2021 were used to identify adults with HPV-associated cancers (cervical, oropharyngeal, vulvar/vaginal, anal/rectal, and penile). Four area based social measures (ABSMs) were used to operationalize neighborhood characteristics: proportion of families living in poverty, proportion of households participating in food stamps, social vulnerability index, and environmental justice index. Distance was measured from the centroid of a person’s census tract to their treatment facility. For each cancer type, we ran four logistic regression models, adjusting for sociodemographic characteristics, to assess associations between distance and each of the ABSMs independently with our outcome, late stage diagnosis.

**Results:**

Among those with cervical cancer, greater distance to diagnosis was associated with later stage at diagnosis, irrespective of ABSM controlled for. Among those with cervical cancer, greater environmental injustice was associated with later stage at diagnosis (OR = 1.78, 95% CI = [1.02,3.09]). Among those with vulvar/vaginal cancer, greater proportion of families in poverty was associated with earlier stage at diagnosis (OR = 0.31, 95% CI = [0.12, 0.78]). There were no interactions between ABSMs and distance to care.

**Conclusions:**

It is important to consider ABSMs and distance to care when identifying those at-risk of late-stage diagnosis.

**Supplementary Information:**

The online version contains supplementary material available at 10.1007/s10552-025-02066-4.

## Introduction

Human papillomavirus (HPV) can cause cancers of the oropharynx, cervix, penis, and anorectal and vulvovaginal areas [1]. The incidence of HPV-associated cancers in the U.S. has increased, with increases in incidence of oropharyngeal, anal, and vulvar cancers between 1999 and 2015 [1]. Given that late-stage diagnosis is associated with lower survival [2], it is imperative to ensure equitable access to diagnostic services, so that increases in incidence are not accompanied by increases in mortality.

Studies pertaining to disparities in healthcare access among those with HPV-related cancers have largely focused on individual-level predictors, such as race, insurance status, marital status, and distance to care [3–7]. Research regarding the association between distance to care and stage at diagnosis among those with HPV-related cancers has produced conflicting findings [3–5], such that distance may [8] or may not [9] be associated with stage at diagnosis, depending on the type of cancer studied.

Neighborhood-level characteristics (often operationalized using area-based social measures (ABSMs)), such as one’s social and built environment, may also be important to cancer-related outcomes including both mortality [10] and stage at diagnosis [11], yet are less often considered. Little is known of the relationship between ABSMs and timely diagnosis of HPV-associated cancers. The few studies that have assessed associations of ABSMs with cancer-related outcomes among HPV-associated cancers have largely focused on poverty only as a predictor of incidence [12, 13] or have grouped all HPV-associated cancers together [11, 14]. These studies have shown that poverty is generally associated with greater incidence and later stage of HPV-associated cancers. Yet, there is a dearth of literature examining the associations of other ABSMs, and other types of HPV-associated cancers and/or outcomes other than incidence. Further, ABSMs may operationalize different concepts related to poverty and social conditions, which may have different relationships with timely diagnosis of HPV-related cancers. Thus, for this exploratory analysis, we chose a selection of ABSMs that had the potential to be informative across different concepts. Inclusion of these ABSMs in our models was ultimately directed by the availability of data and informed by existing literature using a directed acyclic graph approach. Additionally, we chose to assess whether each ABSM independently moderated the association between distance and stage, given that ABSMs may exacerbate the potential association between distance and stage.

Iowa is a unique location to assess associations of ABSMs, distance to care, and cancer-related outcomes, given its rurality, fast growing rates of cancer [15], and highest incidence of oropharyngeal cancer in the nation [16]. Nearly half of Iowans live in rural areas [17] and may be forced to travel further distances for cancer treatment [18], which may compound barriers experienced by those living in areas with less affluent ABSMs. Given conflicting findings regarding the association between distance and stage, as well as the relatively little research conducted regarding ABSMs and stage at diagnosis, our research questions were thus three-fold: among patients living in Iowa with HPV-associated cancer, (1) is distance to place of diagnosis associated with stage at diagnosis independent of ABSM controlled for; (2) are select ABSMs independently associated with stage at diagnosis irrespective of controlling for distance; and (3) does each ABSM independently moderate the association between distance and stage at diagnosis?

## Materials and methods

### Ethics

This study was deemed exempt human subjects research by the Institutional Review Board at the University of Iowa. We received a waiver of informed consent.

### Data sources

We used data from three sources: the Iowa Cancer Registry (ICR), the American Community Survey (ACS), and the Centers for Disease Control and Prevention (CDC) Agency for Toxic Substances and Disease Registry. The ICR has been a part of the National Cancer Institute’s Surveillance, Epidemiology, and End Results Program since 1973 and collects information on all Iowa residents diagnosed with cancer [19]. Data from the ICR was linked by census tract with census tract-level ABSM data from the ACS and the Agency for Toxic Substances and Disease Registry. The ACS [20] collects sociodemographic information from the US population and supplements information collected during the decennial census by the US Census Bureau. For this study, we used the proportion of households participating in the Supplemental Nutrition Assistance Program (SNAP) and proportion of people and families whose level of income was below the federal poverty level during the past 12 months. The Social Vulnerability Index (SVI) and Environmental Justice Index (EJI) were accessed from the Agency for Toxic Substances and Disease Registry [21]. SVI and EJI are place-based scores of communities according to the experienced burden of social factors and environmental factors, respectively. The SVI makes use of 16 US census variables—including housing cost burden, unemployment, and disability status—to describe the social vulnerability of communities [22]. Social vulnerability refers to a community’s susceptibility to being adversely affected by stress (such as disease outbreaks or natural disasters). The EJI ranks census tracts according to 36 different environmental, health, and social factors—including chronic conditions, English proficiency, and measures of air pollution—to describe the effect of environmental injustice on health within each community [23]. We employed four distinct ABSMs as exposures in this study, given that these measures reflect the variety of ways that ABSMs might affect one’s health.

### Sample

Iowa residents diagnosed with microscopically confirmed malignant HPV-associated cancer at age 18 and older between 2010 and 2021 were included in our study sample. HPV-associated cancers were defined according to the CDC definition [24] which includes anal and rectal squamous cell carcinoma (International Classification of Disease for Oncology, Third Edition (ICD-O-3) codes (C21.0–21.8, 20.9)), cervical carcinoma (C53.0–53.9), oropharyngeal squamous cell carcinoma (C01.9, 02.4, 02.8, 05.1–05.2, 09.0–09.1, 09.8–09.9, 10.0–10.4, 10.8–10.9, 14.0, 14.2, 14.8), and vulvar and vaginal squamous cell carcinoma (C51.0–51.9, 52.9). ICD-O-3 histology codes for cervical carcinoma included 8010–8671 and 8940–8941; oropharyngeal squamous cell carcinoma included 8050–8086 and 8120–8131; anal/rectal and vulvar/vaginal squamous cell carcinomas included 8050–8084 and 8120–8131 [24]. We excluded penile squamous cell carcinomas due to small numbers (n = 55). We excluded patients with data collected only by Veterans Administration treatment facilities outside of Iowa, as well as those with information only from the state data exchange, as we were unable to determine the facility providing care to these patients.

### Measures

#### Independent variables

Independent variables of interest included distance to place of diagnosis and the four different ABSMs introduced above.

We calculated distance in miles to place of diagnosis as the distance between the centroid of a patient’s census tract of residence and the reporting facility’s latitude and longitude using Great Circle Distance in ArcGIS Pro 3.2.1 (ESRI, Redlands, CA). This calculation incorporated Universal Transverse Mercator (UTM) Zone 15N, datum NAD83 projection, which represent the mapping projection zone best suited for the longitude of Iowa.

The four ABSM variables were divided into tertiles based on the distribution of the census tracts represented in our sample, such that being placed in a higher tertile indicated living in an area with a greater proportion of households in SNAP, a greater proportion of people in poverty, greater social vulnerability, or greater environmental injustice, respectively (i.e., higher tertile indicates lower socioeconomic status/higher area poverty).

The proportion of households participating in SNAP was not available from ACS from 2010 to 2014. However, for these years, the total number of households participating in SNAP and the total number of households in each census tract was available. Thus, for these years, this measure was manually calculated as ([total number of households participating in SNAP]/[total number of households in census tract]*100), as has been done previously [25]. The variable corresponding to the proportion of households participating in SNAP and the variable corresponding to the proportion of people and families whose level of income was below the federal poverty level during the past 12 months were both linked to each patient according to census tract code and year of diagnosis. Census tract codes from 2010 were used for those diagnosed from 2010 to 2019 and codes from 2020 were used for those diagnosed from 2020 to 2021.

SVI percentile score was available for each census tract for years 2010, 2014, 2016, 2018, and 2020. SVI percentile scores were linked to patients using census tract codes and patient year of diagnosis. We used 2010 census tract codes for those diagnosed prior to 2020 and we used 2020 census tract codes for those diagnosed after 2019. SVI percentile scores were linked according to patient year of diagnosis in the following manner: 2010 census tract-level values were applied to patients living in that tract diagnosed from 2010 to 2013. 2014 census tract-level values were applied to patients living in that tract diagnosed from 2014 to 2015. 2016 census tract-level values were applied to patients living in that tract diagnosed from 2016 to 2017. 2018 census tract-level values were applied to patients living in that tract diagnosed from 2018 to 2019. 2020 census tract-level values were applied to patients living in that tract diagnosed from 2020 to 2021. EJI percentile score was available for 2022 only, and we merged this score to all patients irrespective of year of diagnosis using the 2010 census tract codes [26].

#### Dependent variable

Our primary outcome of interest was whether a cancer was diagnosed at late stage (yes/no); late stage was defined as distant; regional, direct extension and regional lymph nodes; regional, direct extension only; or regional, regional lymph nodes only. These categories were derived using Derived SEER Summary Stage 2000 for diagnosis years 2010–2017 and Derived Summary Stage 2018 for diagnosis years 2018 and beyond [27, 28]. We excluded individuals whose stage was classified as unknown (n = 84).

#### Covariates

Covariates from the ICR included age at diagnosis, race/ethnicity, sex assigned at birth (male or female), marital status, insurance status, rurality at diagnosis, tumor sequence (dichotomized as first primary tumor or not), year of diagnosis, and diagnosing facility type (dichotomized as either not Commission on Cancer (COC) or COC). Given that 95.2% of our sample was non-Hispanic white or white and unknown ethnicity, we dichotomized race as white and other (including Black, American Indian/Alaska Native, Asian/Pacific Islander, Other, and Unknown). Marital status was dichotomized as married/partnered (includes common law/domestic partner) and divorced/separated/widowed/single (never married). Insurance status was divided into five groups: private insurance only, public insurance only, private and public insurance, no insurance, and other/unknown. Rurality at diagnosis was dichotomized as urban (urban commuting area-RUCA codes 1.0, 1.1, 2.0, 2.1, 3.0, 4.1, 5.1, 7.1, 8.1, and 10.1) and rural (all other RUCA codes) using codes released in 2010 [29]. Covariates derived from census tract-level ACS data included median income level in thousands, proportion of census tract unemployed, and proportion of census tract over the age of 25 with at least a high school education. These ACS variables were matched to patient year of diagnosis. Covariates were chosen based on their theoretical association with stage at diagnosis. Given that the EJI and SVI variables are constructed using the ACS variables we employed, we assessed for collinearity in the following ways: we ran logistic regression models containing EJI, SVI, income, employment, and education for each type of cancer. We ensured that the variance inflation factor of all variables in the model was less than 4.0. Additionally, we assessed correlation coefficients between the EJI, SVI, and three ACS variables to ensure that coefficients were less than 0.8.

### Statistical analyses

To describe the dataset, we reported median and quartiles for continuous variables, and relative frequencies for categorical variables. Differences in categorical variables were assessed using the chi-square test or Fisher’s exact test, as appropriate. Normality of continuous variables was evaluated using the Shapiro–Wilk test. For continuous variables, we used the Wilcoxon signed-rank test when data were not normally distributed, and the Student’s t-test when data were normally distributed, to assess differences according to stage and ABSM.

In order to account for potential intra-cluster correlation due to the use of census tract–level data, we chose to apply a generalized estimation equation (GEE) with a logit link function at the census tract level to assess the associations between our independent variables of interest and late-stage cancer diagnosis, stratified by cancer site. We based inference on robust standard errors to ensure validity under possible model misspecification and to account for clustering within census tracts.

For each cancer site, we ran four sets of regression models. Each set of models contained distance and one of the four ABSMs (census tract SVI percentile, census tract EJI percentile, proportion of census tract families living in poverty, or proportion of census tract households participating in SNAP) as our independent variables of interest. We chose not to include all four ABSMs in one model to assess the association between each ABSM and our outcome independent of other ABSMs. Unadjusted models included only the independent variables of interest. The second set of models were fully adjusted for all covariates. Where independent variables of interest were significant in fully adjusted models, we ran a third set of models which included an interaction between distance and the ABSM in that model. If interaction terms were significant, we then planned to compare the interaction model to the fully adjusted model using QIC values, and the best fitting model would be retained as the final model. Models for sex-specific cancers were not adjusted for sex, and oropharyngeal cancer models did not control for race as there were few patients who were non-white (n = 29).

All analyses were conducted in SAS 9.4 software (SAS Institute, Cary, NC). We conducted a complete case analysis. Significance was measured using a cutoff of *p* ≤ 0.05 in all analyses.

## Results

Our sample included 2,227 people, of which 1,048 had oropharyngeal cancer, 483 cervical cancer, 445 anal/rectal cancer, and 251 vulvar/vaginal cancer. Table 1 gives summary statistics for all variables according to stage and cancer type. Among those with oropharyngeal cancer, those with late stage were more likely to be younger (median age = 61 years; *p* = 0.04), male (87%; *p* = 0.01), and living in urban areas (57%; *p* = 0.004). These patients were more likely to be diagnosed with a tumor for the first time (86%; *p* < 0.0001), be treated in a COC-designated facility (54%; *p* = 0.02), and differed from those diagnosed at an early stage according to insurance status (*p* < 0.0001). Among those with cervical cancer, those with late-stage cancer resided farther away from diagnostic site (median distance = 10 miles; *p* = 0.0005), were older (median age = 53 years; *p* < 0.0001), not married (53%; *p* = 0.001), and differed in insurance status from those diagnosed at an early stage (*p* < 0.0001). Among those with anal/rectal cancer, those diagnosed at a late stage were younger (median age = 60 years; *p* = 0.005), female (75%; *p* = 0.01), and differed in insurance status from those diagnosed at an early stage (*p* < 0.0001). Among those with vaginal/vulvar cancer, those diagnosed at early and late stage differed according to insurance status (*p* = 0.0006).

**Table 1:** Full sample demographics of patients diagnosed between 2010–2021 in Iowa according to early and late stage of anal/rectal, cervical, oropharyngeal, vaginal/vulvar cancer, using data from the ICR, ACS, and CDC

Variable	Oropharyngeal		Cervical		Anal/rectal		Vaginal/vulvar	
	Early stage	Late stage	Early stage	Late stage	Early stage	Late stage	Early stage	Late stage
Total (n)	100	948	196	287	212	233	124	127
	Patient-level characteristics							
Age at diagnosis in years*	**63.00 (57.00, 70.00)**	**61.00 (55.00, 68.00)**	**43.00 (37.00, 55.00)**	**53.00 (42.00, 63.00)**	**63.00 (56.00,71.50)**	**60.00 (53.00, 69.00)**	70.00 (57.00, 84.50)	72.00 (56.00, 82.00)
Race/ethnicity Not White	0 (0.00)	29 (3.06)	21 (10.71)	32 (11.15)	9 (4.25)	9 (3.86)	4 (3.23)	3 (2.36)
Sex Female	**22 (22.00)**	**123 (12.97)**	196 (100.00)	287 (100.00)	**136 (64.15)**	**175 (75.11)**	124 (100.00)	127 (100.00)
Marital status Married	55 (55.00)	605 (63.82)	**122 (62.24)**	**136 (47.39)**	111 (52.36)	114 (48.93)	58 (46.77)	46 (36.22)
Rurality Rural	**58 (58.00)**	**407 (42.93)**	80 (40.82)	125 (43.55)	108 (50.94)	113 (48.50)	79 (63.71)	75 (59.06)
Tumor Sequence Not first tumor	**40 (40.00)**	**128 (13.50)**	14 (7.14)	22 (7.67)	47 (22.17)	37 (15.88)	43 (34.68)	30 (23.62)
Year of diagnosis*	**2014.00 (2012.00, 2018.00)**	**2016.00 (2013.00, 2019.00)**	2015.50 (2013.00, 2018.00)	2016.00 (2013.00, 2019.00)	**2016.00 (2013.00, 2018.00)**	**2017.00 (2013.00, 2019.00)**	2015.50 (2013.00, 2019.00)	2016.00 (2013.00, 2019.00)
Diagnosing facility type NCI/COC	**42 (42.00)**	**511 (53.90)**	104 (53.06)	156 (54.36)	95 (44.81)	105 (45.06)	47 (37.90)	56 (44.09)
Insurance Status Public	**32 (32.00)**	**321 (33.86)**	**58 (29.59)**	**110 (38.33)**	**72 (33.96)**	**79 (33.91)**	**40 (32.26)**	**51 (40.16)**
Public & Private	**27 (27.00)**	**176 (18.57)**	**13 (6.63)**	**31 (10.80)**	**63 (29.72)**	**46 (19.74)**	**41 (33.06)**	**42 (33.07)**
None	**2 (2.00)**	**14 (1.48)**	**5 (2.55)**	**19 (6.62)**	**3 (1.42)**	**3 (1.29)**	**1 (0.81)**	**1 (0.79)**
Other	**8 (8.00)**	**68 (7.17)**	**12 (6.12)**	**20 (6.97)**	**14 (6.60)**	**11 (4.72)**	**5 (4.03)**	**3 (2.36)**
	Area-level characteristics							
Distance to place of diagnosis in miles*	14.35 (2.86, 37.35)	10.22 (3.64, 31.77)	**5.18 (2.19, 17.44)**	**9.55 (3.15, 27.90)**	5.30 (1.95, 17.28)	6.40 (2.54, 16.91)	8.75 (2.50, 21.91)	8.51 (1.91, 21.47)
CT median income in thousands*	51.75 (43.80, 65.84)	55.49 (45.70, 68.96)	52.04 (43.58, 60.92)	53.09 (44.35, 61.91)	52.24 (43.51, 61.22)	54.27 (46.04, 64.56)	50.62 (44.63, 59.27)	48.90 (43.14, 58.81)
% of CT unemployed*	4.55 (3.00, 6.85)	4.20 (2.60, 6.20)	4.40 (2.70, 6.25)	4.20 (2.70, 6.80)	4.80 (2.85, 6.90)	4.40 (2.90, 6.80)	4.10 (2.40, 6.55)	4.20 (2.70, 6.50)
% of CT age > 25 & education > HS*	91.80 (89.00, 94.95)	92.70 (88.85, 95.45)	91.50 (87.80, 94.65)	92.40 (88.20, 94.70)	91.60 (88.70, 94.80)	92.30 (88.80, 94.80)	91.25 (88.45, 93.40)	91.60 (88.50, 94.20)
SVI*	0.47 (0.28, 0.74)	0.50 (0.24, 0.76)	0.58 (0.29, 0.81)	0.57 (0.31, 0.83)	0.61 (0.28, 0.80)	0.51 (0.28, 0.78)	0.67 (0.32, 0.81)	0.62 (0.38, 0.82)
EJI*	0.32 (0.18, 0.51)	0.32 (0.14, 0.50)	0.33 (0.17, 0.53)	0.33 (0.18, 0.55)	0.37 (0.19, 0.49)	0.33 (0.20, 0.49)	0.34 (0.20, 0.52)	0.35 (0.19, 0.59)
% below poverty level*	9.95 (7.00, 14.25)	9.70 (5.90, 14.80)	11.05 (7.50, 16.95)	10.60 (6.70, 15.00)	10.30 (7.25, 15.65)	10.40 (7.00, 15.30)	11.95 (8.15, 16.00)	11.20 (6.50, 18.90)
% participating in SNAP*	8.60 (5.43, 12.55)	8.91 (5.40, 14.61)	10.27 (6.28, 17.55)	9.90 (6.40, 16.81)	10.09 (6.12, 16.50)	9.60 (6.00, 15.10)	9.90 (7.30, 15.16)	10.97 (7.00, 19.11)

^*^Column median (quantile range) reported. All other values are column n (%)

Values in bold had significant chi-square, Fisher’s exact test, or t-test results

*CT* census tract, *HS* high school, *NCI* National cancer institute, *COC* commission on cancer, *EJI* environmental just index percentile score, *SVI* social vulnerability index percentile score, *SNAP* supplemental nutrition assistance program

Table 2 gives the results of the multivariable-adjusted logistic regression models for predictors of interest; results of models including information on all covariates are presented in Online Resource 1. All covariates in the adjusted regression models include age at diagnosis, race/ethnicity, sex, marital status, rurality, tumor sequence, diagnosis facility type, and insurance status. Covariates also included census tract-level median income, unemployment rate, and education level. These estimated odds reflect population-averaged associations while accounting for within-tract correlation.

**Table 2:** Adjusted and unadjusted logistic regression models employing odds of being diagnosed at late stage as a function of distance and each ABSM of interest (SVI, EJI, proportion of families and people living below the federal poverty level, and proportion of households participating in SNAP), among those patients diagnosed between 2010 and 2021 in Iowa with anal/rectal, cervical, oropharyngeal, or vaginal/vulvar cancer, using data from the ICR, ACS, and CDC

	SVI percentile score tertiles as predictor							
	Oropharyngeal		Cervical		Anal/Rectal		Vulvar/Vaginal	
SVI	Unadjusted	Adjusted	Unadjusted	Adjusted	Unadjusted	Adjusted	Unadjusted	Adjusted
2nd	0.80 [0.47,1.36]	1.05 [0.57,1.93]	0.91 [0.56,1.47]	1.00 [0.56,1.78]	0.83 [0.52,1.32]	0.93 [0.54,1.60]	1.75 [0.89,3.41]	1.47 [0.70,3.12]
3rd	0.85 [0.50,1.47]	1.30 [0.60,2.82]	0.96 [0.60,1.52]	0.79 [0.39, 1.60]	0.79 [0.51,1.23]	0.88 [0.46,1.68]	1.01 [0.54,1.89]	0.76 [0.32,1.81]
Distance to place of diagnosis	1.00 [0.99,1.00]	1.00 [0.99,1.00]	1.02 [1.01,1.03]***	1.02 [1.01, 1.03]**	1.00 [0.99,1.01]	1.00 [0.99,1.01]	1.00 [0.99,1.01]	1.00 [0.99,1.01]
	EJI percentile score tertiles as predictor							
	Oropharyngeal		Cervical		Anal/Rectal		Vulvar/Vaginal	
EJI	Unadjusted	Adjusted	Unadjusted	Adjusted	Unadjusted	Adjusted	Unadjusted	Adjusted
2nd	0.82 [0.49,1.39]	1.09 [0.57,2.11]	1.49 [0.91,2.43]	1.78 [1.02,3.09]*	1.26 [0.81,1.96]	1.40 [0.85,2.30]	1.22 [0.66,2.27]	1.38 [0.66,2.89]
3rd	1.02 [0.60,1.76]	1.72 [0.83,3.54]	1.33 [0.87,2.04]	1.55 [0.87,2.78]	0.92 [0.59,1.41]	1.16 [0.65,2.07]	1.20 [0.68,2.11]	1.22 [0.55,2.67]
Distance to place of diagnosis	1.00 [0.99,1.00]	1.00 [0.99,1.00]	1.02 [1.01,1.03]***	1.02 [1.01,1.03]**	1.00 [0.99,1.01]	1.00 [0.99,1.01]	1.00 [0.99,1.01]	1.00 [0.99,1.01]
	Proportion of households whose income was below the level of poverty tertiles as predictor							
	Oropharyngeal		Cervical		Anal/Rectal		Vulvar/Vaginal	
Poverty	Unadjusted	Adjusted	Unadjusted	Adjusted	Unadjusted	Adjusted	Unadjusted	Adjusted
2nd	0.86 [0.52,1.44]	1.17 [0.65,2.10]	0.83 [0.52,1.32]	0.90 [0.49,1.65]	0.79 [0.49,1.25]	0.91 [0.52,1.57]	0.49 [0.26,0.93]*	0.31 [0.15,0.65]**
3rd	1.00 [0.59,1.72]	1.86 [0.88,3.95]	1.00 [0.62,1.61]	1.10 [0.50,2.40]	0.85 [0.54,1.34]	1.01 [0.53,1.91]	0.63 [0.35,1.15]	0.31 [0.12,0.78]*
Distance to place of diagnosis	1.00 [0.99,1.00]	1.00 [0.99,1.00]	1.02 [1.01,1.03]***	1.02 [1.01,1.03]**	1.00 [0.99,1.01]	1.00 [0.99,1.01]	1.00 [0.99,1.01]	1.00 [0.99,1.01]
	Proportion of households participating in SNAP tertiles as predictor							
	Oropharyngeal		Cervical		Anal/Rectal		Vulvar/Vaginal	
SNAP	Unadjusted	Adjusted	Unadjusted	Adjusted	Unadjusted	Adjusted	Unadjusted	Adjusted
2nd	0.99 [0.60,1.66]	1.42 [0.82,2.47]	1.23 [0.76,2.01]	1.40 [0.77,2.56]	1.15 [0.71,1.84]	1.26 [0.73,2.17]	0.85 [0.44,1.65]	0.89 [0.45,1.76]
3rd	1.13 [0.66,1.94]	2.04 [0.97,4.29]	1.07 [0.66,1.72]	1.20 [0.59,2.46]	0.86 [0.55,1.33]	0.98 [0.53,1.81]	1.20 [0.65,2.21]	1.13 [0.51,2.50]
Distance to place of diagnosis	1.00[0.99,1.00]	1.00 [0.99,1.01]	1.02 [1.01,1.03]***	1.02 [1.01,1.03]**	1.00 [0.99,1.01]	1.00 [0.99,1.01]	1.00 [0.99,1.01]	1.00 [0.99,1.01]

^*^p < 0.05

^**^p < 0.01

^***^p < 0.001

^a^Reference category of ABSM is 1st tertile. Odds ratios and 95% confidence intervals reported (OR [95% CI])

^b^*EJI* environmental just index percentile score, *SVI* social vulnerability index percentile score, *SNAP* supplemental nutrition assistance program

^c^Unadjusted models include one of the four ABSMs (EJI, SVI, Poverty, SNAP) and distance to place of diagnosis as predictors. Adjusted models include those variables in the unadjusted models as well as covariates

### Oropharyngeal cancer

In models using distance and one of the four ABSMs as independent variables, none of these terms were significant in either adjusted or unadjusted models.

### Cervical cancer

In unadjusted models using distance and SVI, greater distance was associated with late-stage diagnosis (odds ratio (OR) = 1.02, 95% confidence interval (CI) = [1.01,1.03]) and this association remained in the fully-adjusted model (adjusted odds ratio (aOR) = 1.02, 95% CI = [1.01,1.03]). Neither SVI, nor the interaction between SVI and distance were significant.

In unadjusted models using distance and EJI, greater distance was associated with late-stage diagnosis (OR = 1.02, 95% CI = [1.01,1.03]) and this association remained in the fully-adjusted model (aOR = 1.02, 95% CI = [1.01,1.03]). In the fully-adjusted model, those with EJI scores in the second tertile (greater environmental burden) were more likely to be diagnosed at a later stage than those in the first tertile (lower environmental burden; aOR = 1.78, 95% CI = [1.02,3.09]). The interaction between EJI and distance was not significant.

In unadjusted models using distance and proportion of people and families whose level of income was below the federal poverty level during the past 12 months, greater distance was associated with late-stage diagnosis (OR = 1.02, 95% CI = [1.01,1.03]) and this association remained in the fully-adjusted model (aOR = 1.02, 95% CI = [1.01,1.03]). Neither proportion of people and families whose level of income was below the federal poverty level during the past 12 months, nor the interaction between this variable and distance were significant.

In models using distance and proportion of households participating in SNAP, greater distance was associated with late-stage diagnosis (OR = 1.02, 95% CI = [1.01,1.03]) and this association remained in the fully-adjusted model (aOR = 1.02, 95% CI = [1.01,1.03]). Proportion of households participating in SNAP was not significant. The interaction between SNAP participation and distance was not significant.

### Anal/rectal cancer

In models using distance and one of the four ABSMs as independent variables, none of these terms were significant in either adjusted or unadjusted models.

### Vulvar/vaginal cancer

In models using distance and SVI, distance was not significantly associated with late stage diagnosis. In the unadjusted model, those in the third tertile (greater social vulnerability) were less likely than those in the second (lower social vulnerability) to present at late-stage diagnosis (OR = 0.57, 95% CI = [0.33,0.99]; results not shown). This association remained in the fully-adjusted model (aOR = 0.52, 95% CI = [0.27,0.99]; results not shown). The interaction between SVI and distance was not significant.

In models using distance and EJI, neither was associated with late-stage diagnosis in unadjusted or adjusted models.

In models using distance and proportion of people and families whose level of income was below the federal poverty level during the past 12 months, distance was not significantly associated with late stage diagnosis. In the unadjusted model, those in the second tertile (greater number of families in poverty) were less likely than those in the first (fewer number of families in poverty) to present at late-stage diagnosis (OR = 0.49, 95% CI = [0.26,0.93]). In the fully-adjusted model, those in the second tertile (aOR = 0.31, 95% CI = [0.15,0.65]) and those in the third tertile (aOR = 0.31, 95% CI = [0.12,0.78]) were less likely than those in the first tertile to present at a late-stage. The interaction between poverty and distance was not significant.

In models using distance and proportion of households participating in SNAP, neither term was significantly associated with late-stage diagnosis in either unadjusted or adjusted models.

## Discussion

In this sample of Iowan patients, greater distance was associated with slightly increased odds of late-stage diagnosis among those with certain HPV-related cancers. However, while significant, the relative weakness of this association suggests this relationship may not be highly clinically relevant. Additionally, there were few associations between ABSMs and a later stage at diagnosis, and many associations were weak, if statistically significant. These largely null associations could support a “true” lack of association or could reflect limitations of census-tract level ABSMs, which do not reflect individual-level household socioeconomic status (i.e., may be subject to ecological fallacy). Collectively, our findings suggest that distance to diagnosis may have slight implications for stage at diagnosis, depending on HPV-related cancer type. However, among Iowans with HPV-associated cancer, ABSMs may not be a strong predictor of stage at diagnosis.

Overall, while the association between distance and late stage at diagnosis appears to be positive, the literature indicates that it varies according to cancer type and the state in which the study was conducted [3–5, 30]. Similarly, among Iowans, this association is less clear, and, as in this study, varied according to cancer site [8, 9]. For example, in our study, this association existed only among those with cervical cancer and was relatively weak. One reason for the variability in the association between distance to diagnosis and stage at diagnosis in our results may be related to the fact that cervical cancer is the only type of cancer among those we studied for which there are screening guidelines [31]. For example, there may be an element of screening fatigue such that patients with greater distance to care are less likely to complete screenings that are required more frequently [32]. This idea is supported by the fact that a similar effect of distance was also seen in studies assessing the use of mammography to screen for breast cancer [8], but not colonoscopy [9]. An alternative theory was posited by Charlton and colleagues [9] who identified a null association between travel time and being diagnosed with a later stage of colorectal cancer. They found that roughly a quarter of their sample traveled past the nearest colonoscopy provider to obtain care. This suggests that other factors, such as referral patterns or one’s familiarity of a physician, may play a role in where patients in Iowa decide to obtain care beyond distance. Because there is no clear relationship, more research is needed to elucidate treatment patterns among rural residents and those living further from care.

Our findings of differing associations between ABSM and late-stage diagnosis according cancer site may reflect the variety of factors that can influence healthcare access, utilization, and quality captured by the multiple ABSMs. For example, the SVI [22] employs factors including access to transportation, which may influence one’s ability to obtain care [33], as well as level of education and English language proficiency, which may impact whether one engages in preventive care [34]. In contrast, the EJI encompasses aspects of social vulnerability, health vulnerability, and environmental burden [26]. Environmental justice pertains to the idea that all people in a community should have equal opportunities to be involved in the decisions that impact the health of their environment [35]. The stressors associated with living in an environment with a greater environmental burden may limit the “mental bandwidth” one has to address health-related issues [36], and may be seen in our sample of Iowans with cervical cancer. Finally, the proportion of families living in poverty and/or participating in SNAP may indicate a community’s ability to meet their basic needs. Those experiencing competing demands may need to address certain basic needs over healthcare-related needs such as cancer screening [37–39].

Our study had strengths and limitations that should be considered in its interpretation. Given the small number of patients with penile cancer in our study, we were unable to include them in our analyses. This is unfortunate, given that this cancer is relatively understudied [40–42]. Another limitation of our analysis is that the data were unbalanced, with many census tracts containing only a single participant. This limited our ability to specify a more complex working correlation structure in the GEE models as opposed to an independent correlation structure. While GEE provides consistent estimates even under misspecification of the correlation structure, using an independent structure in the presence of within-cluster correlation can lead to conservative standard errors and attenuated effect estimates, which may account for our largely null findings [43, 44]. An additional limitation includes our use of census tract centroid in calculating distance to diagnosis, rather than population-weighted centroid, which may more accurately capture distance. Furthermore, while employing “as the crow fly” distances rather than actual driving distance has limitations, studies have shown that these distances are relatively comparable [45, 46]. Additionally, the use of one year of EJI data may not accurately reflect true EJI scores for all years of diagnosis included in our sample. Furthermore, by employing a complete case analysis, we may have introduced selection bias. Specifically, we found differences in insurance status, tumor sequence, rurality, marital status, sex, diagnosing facility type, stage, income, and age between our included sample and those excluded due to missing date. Lastly, given the exploratory nature of our study, we chose to accept the increased risk of a Type I error occurring, with the understanding that future studies are needed to confirm our findings. Strengths of this study included assessment of four different ABSMs, whereas prior literature has largely focused on poverty or individual-level characteristics including insurance status or race/ethnicity and their association with cancer-related outcomes [6, 11, 14]. This provides a better understanding of how different ABSMs might influence the stage at which a person is diagnosed with cancer.

## Conclusion

Collectively, our findings show the differential effects that distance to place of diagnosis and ABSMs may have on stage at diagnosis, according to cancer type. This information is important for developing policies aimed at improving outcomes for those most vulnerable. Our study also emphasizes the value of collecting individual-level data on ABSMs through state cancer registries.

## Supplementary Information

Below is the link to the electronic supplementary material.

Supplementary file1 (DOCX 42 KB)

## Data Availability

Iowa Cancer Registry data are available through reasonable request to registry investigators, or directly through the SEER Program. Data from the ACS are publicly available: https://www.census.gov/programs-surveys/acs/data.html Data on the CDC’s Social Vulnerability Index and Environmental Justice Index were previously publicly available and are available from the corresponding author on reasonable request.
