# Supplementary material for: Association of distance to diagnosis and area-based social measures with stage at diagnosis among Iowans with HPV-related cancers
Source: Cancer Causes Control. 2025 Sep 10;36(12):1881–90. doi: 10.1007/s10552-025-02066-4 (PMC12630225; doi:10.1007/s10552-025-02066-4)
Supplement: Supplementary file 1 — Supplementary file1 (DOCX 42 KB) [file 10552_2025_2066_MOESM1_ESM.docx]

**Article Title:** Association of Distance to Diagnosis and Area-Based Social Measures with Stage at Diagnosis Among Iowans with HPV-Related Cancers

**Journal Name:** Cancer Causes & Control

**Author Names:** Emily Janio MPH, Amanda R Kahl MPH, Dosten Kpozehouen MSc MPH, Natoshia Askelson MPH PhD, Sarah H Nash MPH PhD

**Corresponding Author:** Emily Janio, [emily-janio@uiowa.edu](mailto:emily-janio@uiowa.edu), University of Iowa

Supplemental Material. Adjusted and unadjusted logistic regression models employing odds of being diagnosed at late stage as a function of distance and each ABSM of interest (SVI, EJI, proportion of families and people living below the federal poverty level, and proportion of households participating in SNAP), among those patients diagnosed between 2010-2021 in Iowa with anal/rectal, cervical, oropharyngeal, or vaginal/vulvar cancer, using data from the ICR, ACS, and CDC

|  | **Oropharyngeal** | | **Cervical** | | **Anal/Rectal** | | **Vulvar/Vaginal** | |
| --- | --- | --- | --- | --- | --- | --- | --- | --- |
| SVI | **Unadjusted** | **Adjusted** | **Unadjusted** | **Adjusted** | **Unadjusted** | **Adjusted** | **Unadjusted** | **Adjusted** |
| 2^nd^ | 0.80 [0.47,1.36] | 1.05 [0.57,1.93] | 0.91 [0.56,1.47] | 1.00 [0.56,1.78] | 0.83 [0.52,1.32] | 0.93 [0.54,1.60] | 1.75 [0.89,3.41] | 1.47 [0.70,3.12] |
| 3^rd^ | 0.85 [0.50,1.47] | 1.30 [0.60,2.82] | 0.96 [0.60,1.52] | 0.79 [0.39, 1.60] | 0.79 [0.51,1.23] | 0.88 [0.46,1.68] | 1.01 [0.54,1.89] | 0.76 [0.32,1.81] |
| Distance to place of diagnosis | 1.00 [0.99,1.00] | 1.00 [0.99,1.00] | 1.02 [1.01,1.03]*** | 1.02 [1.01, 1.03]** | 1.00 [0.99,1.01] | 1.00 [0.99,1.01] | 1.00 [0.99,1.01] | 1.00 [0.99,1.01] |
| Age at diagnosis |  | 0.99 [0.97,1.02] |  | 1.05 [1.03, 1.07]*** |  | 0.99 [0.96,1.01] |  | 0.98 [0.96,1.00] |
| Insurance (Private)  None |  | 0.55 [0.10,2.92] |  | 3.52 [1.04,11.90]* |  | 0.63 [0.12,3.21] |  | 0.74 [0.031,17.70] |
| Other |  | 0.88 [0.32,2.42] |  | 1.70 [0.77,3.76] |  | 0.53 [0.23,1.21] |  | 0.71 [0.14,3.53] |
| Public |  | 1.38 [0.76,2.50] |  | 1.59 [0.97,2.61] |  | 0.83 [0.48,1.44] |  | 1.74 [0.67,4.24] |
| Public & Private |  | 0.90 [0.46,1.76] |  | 0.79 [0.34,1.82] |  | 0.64 [0.34,1.24] |  | 1.68 [0.70,4.07] |
| % of CT age >25 & education >HS |  | 1.00 [0.95,1.05] |  | 0.99 [0.96,1.02] |  | 0.99 [0.95,1.03] |  | 0.99 [0.94,1.05] |
| % of CT unemployed |  | 0.96 [0.90,1.03] |  | 1.03 [0.96,1.10] |  | 1.00 [0.93,1.08] |  | 0.97 [0.87,1.07] |
| CT median income in thousands |  | 1.00 [0.99,1.02] |  | 1.01 [0.99,1.03] |  | 1.01 [0.99,1.02] |  | 0.99 [0.96,1.01] |
| Diagnosing facility type (Not NCI/COC)  NCI/COC |  | 1.47 [0.89,2.44] |  | 0.81 [0.50,1.32] |  | 0.93 [0.56,1.53] |  | 1.60 [0.79,3.25] |
| Sex (Male)  Female |  | 0.64 [0.35,1.15] |  |  |  | 1.67 [1.09,2.57]* |  |  |
| Marital status (Not married)  Married |  | 1.56 [0.96,2.54] |  | 0.58 [0.38,0.89]* |  | 0.89 [0.60,1.33] |  | 0.63 [0.35,1.12] |
| Race/ethnicity (White)  Not white |  |  |  | 1.03 [0.55,1.93] |  | 0.86 [0.34,2.16] |  | 0.73 [0.12,4.57] |
| Rurality (Urban)  Rural |  | 0.64 [0.39,1.07] |  | 0.98 [0.58,1.65] |  | 0.95 [0.58,1.55] |  | 0.95 [0.46,1.98] |
| Tumor Sequence (First tumor)  Not first tumor |  | 0.22 [0.14,0.37]*** |  | 0.52 [0.21,1.30] |  | 0.76 [0.46,1.26] |  | 0.55 [0.31,0.98]* |

|  | **Oropharyngeal** | | **Cervical** | | **Anal/Rectal** | | **Vulvar/Vaginal** | |
| --- | --- | --- | --- | --- | --- | --- | --- | --- |
| EJI | **Unadjusted** | **Adjusted** | **Unadjusted** | **Adjusted** | **Unadjusted** | **Adjusted** | **Unadjusted** | **Adjusted** |
| 2^nd^ | 0.82 [0.49,1.39] | 1.09 [0.57,2.11] | 1.49 [0.91,2.43] | 1.78 [1.02,3.09]* | 1.26 [0.81,1.96] | 1.40 [0.85,2.30] | 1.22 [0.66,2.27] | 1.38 [0.66,2.89] |
| 3^rd^ | 1.02 [0.60,1.76] | 1.72 [0.83,3.54] | 1.33 [0.87,2.04] | 1.55 [0.87,2.78 ] | 0.92 [0.59,1.41] | 1.16 [0.65,2.07] | 1.20 [0.68,2.11] | 1.22 [0.55,2.67] |
| Distance to place of diagnosis | 1.00 [0.99,1.00] | 1.00 [0.99,1.00] | 1.02 [1.01,1.03]*** | 1.02 [1.01,1.03]** | 1.00 [0.99,1.01] | 1.00 [0.99,1.01] | 1.00 [0.99,1.01] | 1.00 [0.99,1.01] |
| Age at diagnosis |  | 0.99 [0.97,1.02] |  | 1.05 [1.03,1.07]*** |  | 0.99 [0.97,1.01] |  | 0.98 [0.96,1.00] |
| Insurance (Private)  None |  | 0.57 [0.11,3.06] |  | 3.53 [1.08,11.52]* |  | 0.64 [0.13,3.26] |  | 0.96 [0.069,13.38] |
| Other |  | 0.86 [0.31,2.37] |  | 1.76 [0.78,3.93] |  | 0.54 [0.24,1.23] |  | 0.72 [0.15,3.42] |
| Public |  | 1.38 [0.77,2.49] |  | 1.53 [0.93,2.53] |  | 0.81 [0.47,1.40] |  | 1.98 [0.90,4.36] |
| Public & Private |  | 0.90 [0.46,1.76] |  | 0.77 [0.34,1.77] |  | 0.62 [0.33,1.20] |  | 1.85 [0.74,4.63] |
| % of CT age >25 & education >HS |  | 1.00 [0.96,1.05] |  | 1.00 [0.97,1.03] |  | 1.00 [0.96,1.03] |  | 1.01 [0.95,1.07] |
| % of CT unemployed |  | 0.96 [0.89,1.03] |  | 1.02 [0.96,1.09] |  | 1.00 [0.93,1.08] |  | 0.96 [0.87,1.06] |
| CT median income in thousands |  | 1.01 [0.99,1.02] |  | 1.02 [1.00,1.04] |  | 1.01 [0.99,1.02] |  | 0.99 [0.96,1.02] |
| Diagnosing facility type (Not NCI/COC)  NCI/COC |  | 1.43 [0.86,2.37] |  | 0.83 [0.51,1.36] |  | 0.92 [0.56,1.52] |  | 1.57 [0.79,3.13] |
| Sex (Male)  Female |  | 0.67 [0.37,1.21] |  |  |  | 1.68 [1.10,2.58]* |  |  |
| Marital status (Not married)  Married |  | 1.59 [0.98,2.56] |  | 0.59 [0.39,0.90]* |  | 0.88 [0.59,1.31] |  | 0.65 [0.37,1.14] |
| Race/ethnicity (White)  Not white |  |  |  | 1.04 [0.57,1.91] |  | 0.85 [0.34,2.15] |  | 0.66 [0.12,3.55] |
| Rurality (Urban)  Rural |  | 0.59 [0.34,1.00] |  | 0.91 [0.53,1.55] |  | 0.90 [0.54,1.51] |  | 0.86 [0.41,1.79] |
| Tumor Sequence (First tumor)  Not first tumor |  | 0.23 [0.14,0.38]*** |  | 0.50 [0.20,1.25] |  | 0.77 [0.46,1.27] |  | 0.57 [0.32,1.02] |

|  | **Oropharyngeal** | | **Cervical** | | **Anal/Rectal** | | **Vulvar/Vaginal** | |
| --- | --- | --- | --- | --- | --- | --- | --- | --- |
| Poverty | **Unadjusted** | **Adjusted** | **Unadjusted** | **Adjusted** | **Unadjusted** | **Adjusted** | **Unadjusted** | **Adjusted** |
| 2^nd^ | 0.86 [0.52,1.44] | 1.17 [0.65,2.10] | 0.83 [0.52,1.32] | 0.90 [0.49,1.65] | 0.79 [0.49,1.25] | 0.91 [0.52,1.57] | 0.49 [0.26,0.93]* | 0.31 [0.15,0.65]** |
| 3^rd^ | 1.00 [0.59,1.72] | 1.86 [0.88,3.95] | 1.00 [0.62,1.61] | 1.10 [0.50,2.40] | 0.85 [0.54,1.34] | 1.01 [0.53,1.91] | 0.63 [0.35,1.15] | 0.31 [0.12,0.78]* |
| Distance to place of diagnosis | 1.00 [0.99,1.00] | 1.00 [0.99,1.00] | 1.02 [1.01,1.03]*** | 1.02 [1.01,1.03]** | 1.00 [0.99,1.01] | 1.00 [0.99,1.01] | 1.00 [0.99,1.01] | 1.00 [0.99,1.01] |
| Age at diagnosis |  | 0.99 [0.97,1.02] |  | 1.05 [1.03,1.07]*** |  | 0.99 [0.96,1.01] |  | 0.98 [0.96,1.00] |
| Insurance (Private)  None |  | 0.60 [0.11,3.32] |  | 3.41 [1.02,11.38]* |  | 0.64 [0.12,3.29] |  | 0.92 [0.060,13.95] |
| Other |  | 0.89 [0.32,2.44] |  | 1.66 [0.76,3.66] |  | 0.52 [0.23,1.19] |  | 0.62 [0.12,3.23] |
| Public |  | 1.37 [0.76,2.45] |  | 1.56 [0.95,2.56] |  | 0.83 [0.48,1.42] |  | 1.62 [0.73,3.61] |
| Public & Private |  | 0.91 [0.46,1.77] |  | 0.79 [0.34,1.83] |  | 0.64 [0.34,1.23] |  | 1.61 [0.64,4.05] |
| % of CT age >25 & education >HS |  | 1.00 [0.95,1.05] |  | 1.00 [0.97,1.03] |  | 1.00 [0.96,1.03] |  | 1.00 [0.95,1.06] |
| % of CT unemployed |  | 0.95 [0.89,1.02] |  | 1.02 [0.95,1.09] |  | 1.00 [0.93,1.08] |  | 0.97 [0.87,1.08] |
| CT median income in thousands |  | 1.01 [0.99,1.03] |  | 1.01 [0.99,1.03] |  | 1.01 [0.99,1.02] |  | 0.97 [0.94,1.00]* |
| Diagnosing facility type (Not NCI/COC)  NCI/COC |  | 1.47 [0.89,2.45] |  | 0.79 [0.49,1.30] |  | 0.93 [0.56,1.53] |  | 1.58 [0.80,3.14] |
| Sex (Male)  Female |  | 0.63 [0.34,1.15] |  |  |  | 1.68 [1.09,2.58]* |  |  |
| Marital status (Not married)  Married |  | 1.57 [0.97,2.53] |  | 0.59 [0.38,0.90]* |  | 0.89 [0.60,1.33] |  | 0.60 [0.33,1.07] |
| Race/ethnicity (White)  Not white |  |  |  | 1.05 [0.56,1.96] |  | 0.85 [0.34,2.15] |  | 0.80 [0.14,4.46] |
| Rurality (Urban)  Rural |  | 0.67 [0.40,1.11] |  | 0.98 [0.58,1.66] |  | 0.96 [0.58,1.59] |  | 0.90 [0.44,1.85] |
| Tumor Sequence (First tumor)  Not first tumor |  | 0.23 [0.14,0.37]*** |  | 0.50 [0.20,1.26] |  | 0.76 [0.46,1.26] |  | 0.49 [0.27,0.89]* |

|  | **Oropharyngeal** | | **Cervical** | | **Anal/Rectal** | | **Vulvar/Vaginal** | |
| --- | --- | --- | --- | --- | --- | --- | --- | --- |
| SNAP | **Unadjusted** | **Adjusted** | **Unadjusted** | **Adjusted** | **Unadjusted** | **Adjusted** | **Unadjusted** | **Adjusted** |
| 2^nd^ | 0.99 [0.60,1.66] | 1.42 [0.82,2.47] | 1.23 [0.76,2.01] | 1.40 [0.77,2.56] | 1.15 [0.71,1.84] | 1.26 [0.73,2.17] | 0.85 [0.44,1.65] | 0.89 [0.45,1.76] |
| 3^rd^ | 1.13 [0.66,1.94] | 2.04 [0.97,4.29] | 1.07 [0.66,1.72] | 1.20 [0.59,2.46] | 0.86 [0.55,1.33] | 0.98 [0.53,1.81] | 1.20 [0.65,2.21] | 1.13 [0.51,2.50] |
| Distance to place of diagnosis | 1.00[0.99,1.00] | 1.00 [0.99,1.00] | 1.02 [1.01,1.03]*** | 1.02 [1.01,1.03]** | 1.00 [0.99,1.01] | 1.00 [0.99,1.01] | 1.00 [0.99,1.01] | 1.00 [0.99,1.01] |
| Age at diagnosis |  | 0.99 [0.97,1.02] |  | 1.05 [1.03,1.07]*** |  | 0.98 [0.96,1.01] |  | 0.98 [0.96,1.00] |
| Insurance (Private)  None |  | 0.59 [0.10,3.34] |  | 3.61 [1.09,11.90]* |  | 0.65 [0.13,3.29] |  | 0.80 [0.048,13.26] |
| Other |  | 0.90 [0.33,2.43] |  | 1.76 [0.80,3.90] |  | 0.54 [0.24,1.23] |  | 0.82 [0.17,3.90] |
| Public |  | 1.37 [0.76,2.46] |  | 1.61 [0.98,2.65] |  | 0.83 [0.48,1.43] |  | 1.91 [0.89,4.13] |
| Public & Private |  | 0.94 [0.48,1.83] |  | 0.85 [0.37,1.96] |  | 0.64 [0.33,1.23] |  | 1.74 [0.70,4.32] |
| % of CT age >25 & education >HS |  | 1.00 [0.96,1.05] |  | 1.00 [0.97,1.03] |  | 0.99 [0.96,1.03] |  | 1.01 [0.95,1.06] |
| % of CT unemployed |  | 0.96 [0.89,1.02] |  | 1.02 [0.96,1.09] |  | 1.01 [0.93,1.09] |  | 0.96 [0.87,1.06] |
| CT median income in thousands |  | 1.01 [0.99,1.03] |  | 1.01 [0.99,1.04] |  | 1.01 [0.99,1.02] |  | 0.99 [0.96,1.02] |
| Diagnosing facility type (Not NCI/COC)  NCI/COC |  | 1.45 [0.87,2.43] |  | 0.81 [0.49,1.32] |  | 0.91 [0.55,1.51] |  | 1.53 [0.75,3.10] |
| Sex (Male)  Female |  | 0.67 [0.36,1.22] |  |  |  | 1.68 [1.09,2.57]* |  |  |
| Marital status (Not married)  Married |  | 1.57 [0.97,2.54] |  | 0.59 [0.38,0.90]* |  | 0.88 [0.59,1.31] |  | 0.66 [0.37,1.15] |
| Race/ethnicity (White)  Not white |  |  |  | 1.03 [0.56,1.92] |  | 0.89 [0.35,2.26] |  | 0.65 [0.12,3.56] |
| Rurality (Urban)  Rural |  | 0.66 [0.39,1.10] |  | 0.97 [0.57,1.65] |  | 0.91 [0.55,1.51] |  | 0.91 [0.44,1.87] |
| Tumor Sequence (First tumor)  Not first tumor |  | 0.22 [0.13, 0.36]*** |  | 0.52 [0.21,1.31] |  | 0.77 [0.46,1.28] |  | 0.57 [0.32,1.01] |

*p<0.05

**p<0.01

***p<0.001

^a^Reference category of ABSM is 1^st^ tertile. Odds ratios and 95% confidence intervals reported (OR [95% CI])

^b^Abbreviations: Environmental just index percentile score (EJI), Social vulnerability index percentile score (SVI), Supplemental Nutrition Assistance Program (SNAP)

^c^Unadjusted models include one of the four ABSMs (EJI, SVI, Poverty, SNAP) and distance to place of diagnosis as predictors. Adjusted models include those variables in the unadjusted models as well as covariates.
